# Intracranial lesion as onset symptom in a patient with early undifferentiated connective tissue disease: a case report

**DOI:** 10.1186/s12883-017-0868-4

**Published:** 2017-05-05

**Authors:** Ying Du, Chuan Li, Dai-di Zhao, Jia-rui Lu, Wei Zhang, Zhu-yi Li

**Affiliations:** Department of Neurology, Tangdu Hospital, Fourth Military Medical University, Xi’an City, Shaanxi Province 710038 China

**Keywords:** Intracranial lesion, Undifferentiated connective tissue disease

## Abstract

**Background:**

Undifferentiated connective tissue disease (UCTD) is widely considered to be a distinct clinical entity, and now divided into two subgroups: stable UCTD and early UCTD. The most frequent onset symptoms of UCTD include arthralgias, arthritis, Raynaud’s phenomenon, mucocutaneous involvement, and sicca symptoms. However, Neurologic involvement is rare, and intracranial lesion as onset symptom in a patient with early UCTD has not yet been reported.

**Case presentation:**

A 51-year-old Chinese female experienced progressive left leg weakness for 14 days before hospitalizing in our department. The lesion on right parietal lobe was initially detected by brain magnetic resonance imaging. Although the patient declined a cerebral biopsy, the possibility of stroke, cerebral venous sinus thrombosis, NMOSD, MS, autoimmune encephalitis, intracranial infections, and malignant tumors as cause of the lesion was excluded by intracranial angiogram, CSF study, MRI enhancement and MRS examination. Moreover, immunologic studies showed high titer of antinuclear antibody, increased erythrocyte sedimentation rate and C-reactive protein. These results led to a diagnosis of early UCTD with central nerve system (CNS) involvement. After low dose corticosteroid and azathioprine therapy, the patient’s symptoms, abnormalities in immunologic tests and cerebral radiologic examinations were all greatly improved within a short duration.

**Conclusions:**

This is the first report of intracranial lesion as onset symptom in a patient with early UCTD. Our case suggested that central nerve system (CNS) involvement could be the onset symptom in early UCTD, and should be recognized quickly with exclusion of other causative factors in the differential diagnosis. Prompt and adequate treatment with low-dose steroid and immunosuppressive drugs could improve the prognosis of both early UCTD and CNS involvement.

**Electronic supplementary material:**

The online version of this article (doi:10.1186/s12883-017-0868-4) contains supplementary material, which is available to authorized users.

## Background

The term undifferentiated connective tissue disease (UCTD) refers to unclassifiable systemic autoimmune diseases, which share some clinical and serological manifestations with definite connective tissue diseases (CTDs) but not fulfilling any of the existing classification criteria for certain CTDs, such as systemic lupus erythematosus (SLE), systemic sclerosis (SSc), Sjögren syndrome (SS), dermatomyositis/polymyositis (DM/PM), mixed connective tissue diseases (MCTD) and rheumatoid arthritis (RA) [1]. It is now widely accepted that UCTD represent a distinct clinical entity including two subgroups: stable UCTD and early UCTD. Among these UCTD patients, severe organ involvements (such as heart, renal or neurological manifestations) are rarely reported [2–4]. Here, we firstly described a female early UCTD patient with intracranial lesions as the onset and main clinical manifestations in order to improve the diagnosis and treatment of the disease.

## Case presentation

A 51-year-old Chinese female experienced progressive left leg weakness for 14 days before hospitalizing in our department. At the onset of the symptom, the patient presented dizziness and left lower limb weakness, and the symptom was still progressive, even after treatment as brain ischemia. Then the patient was transferred to our department. On admission, the physical examinations were all normal, and neurologic examination disclosed that proximal and distal muscle strength of left lower limb was 3/5 by manual muscles testing (MTT). The superficial and deep sensory examinations were completed by cotton swab, tuning fork and pinprick, and all sensory modalities and tendon reflexes were normal, and pathologic reflexes were negative. Cranial nerve function and the mental examination were normal. No family history of neurological disease and metabolic-related disorders had been previously documented, according to her medical records.

Blood analysis showed erythrocyte sedimentation rate of 40 mm/h (normal <20 mm/h) and C-reactive protein of 55.1 mg/L (normal <3.0 mg/L). In the tests for autoantibody, the antinuclear antibody (ANA) was positive, and the titer was greatly elevated to 1:10,000. But, antibodies to double stranded DNA, Sm, Ro, La, Scl-7, Jo-1, ACA, ribonucleoprotein, nucleosomes, and histone were negative. Rheumatoid factor, anti-CCP antibody, and ANCA were also negative. The values for blood glucose, liver function, renal function, electrolytes, serum folate, vitamin B12, homocysteine, lactic acid, tumor markers, thyroid function and associated antibodies, C3, and C4 were their normal ranges. Tests for virus, syphilis, HIV, NMOSD, MS (Additional file 1) and autoimmune encephalitis were all negative.

Lumbar puncture was performed and showed a normal intracranial pressure; the biochemical and hematologic tests of the cerebrospinal fluid (CSF) revealed a normal glucose, chloride and protein level; the white cell count was elevated 27 × 106/L with increasing percentage of lymphocyte 77.8%; and the CSF cultures for bacteria, viruses, and fungi were all negative.

**Fig. 1:**
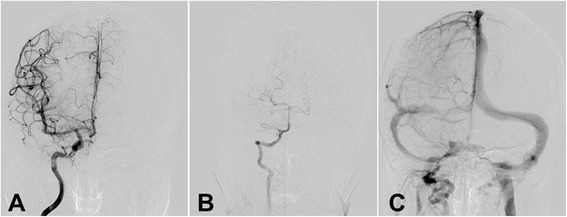
Intracranial angiogram on 3rd hospital day before treatment showed that *right* internal carotid artery **a**, *right* vertebral artery and basilar artery **b**, and all venous sinuses **c** were normal

**Fig. 2:**
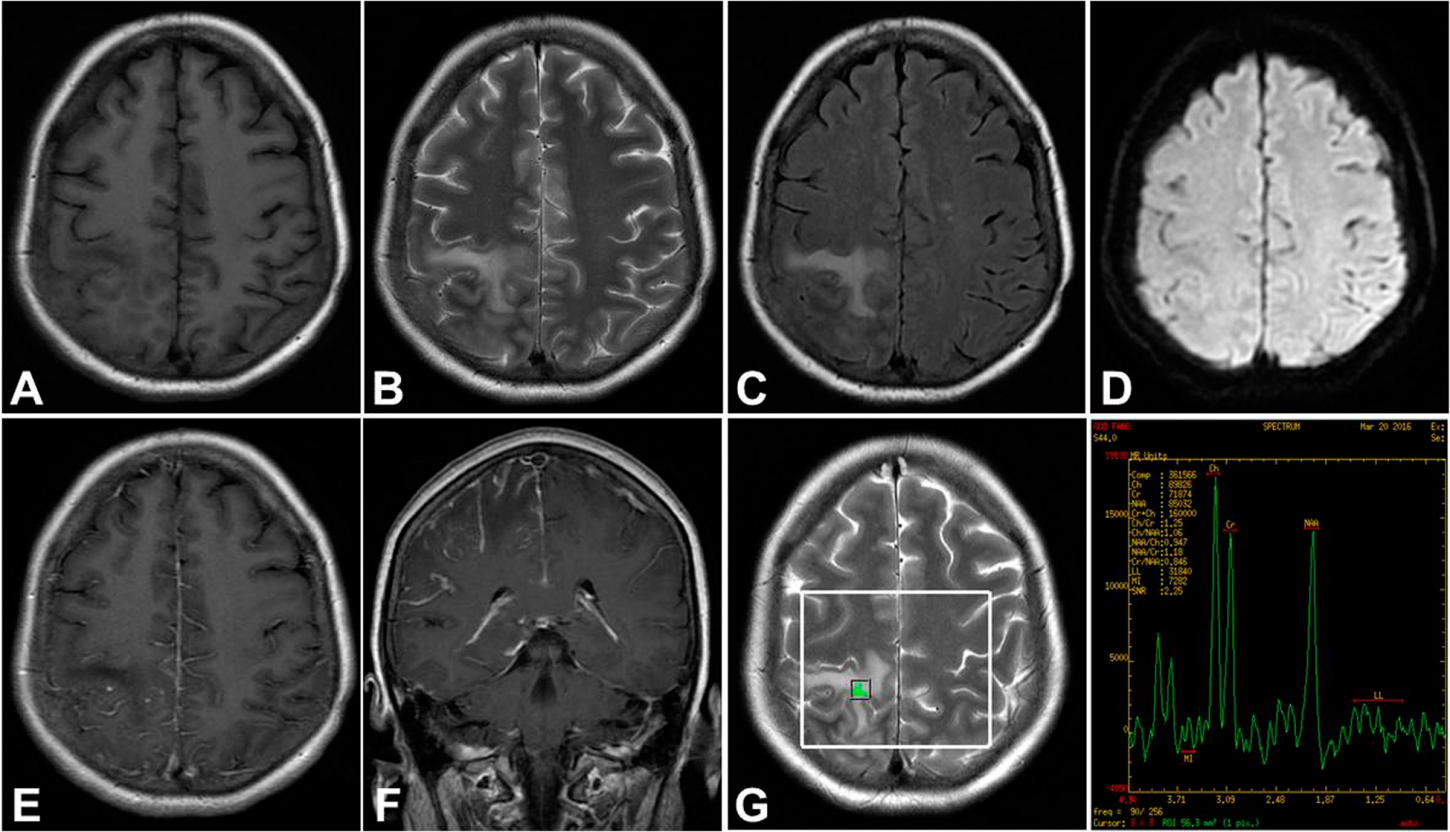
MRI on the 1st hospital day showed a lesion on *right* parietal lobe that was hypointensity on T1-weighted (**a**), hyperintensity on T2-weighted images (**b**), high signal on FLAIR (**c**), and normal signal on DWI (**d**). MRI enhancement showed contrast enhancement along the surface of swollen gyrus lesion (**e**) and (**f**). MRS showed slightly decreased NAA and increased Cr and Cho peak on the lesion (**g**)

Intracranial angiogram was normal (Fig. 1). Brain magnetic resonance imaging (MRI) revealed a lesion on right parietal lobe that was hypointensity on T1-weighted, hyperintensity on T2-weighted images, and high signal on FLAIR. There was slim contrast enhancement along the surface of swollen gyrus lesion after Gd-DTPA injection (10 mL/kg of body weight). MRS showed slightly decreased NAA and increased Cr and Cho peak on the right parietal lobe lesion compared to same region on normal left side (Fig. 2). Chest CT showed normal. The patient declined a cerebral biopsy.

**Fig. 3:**
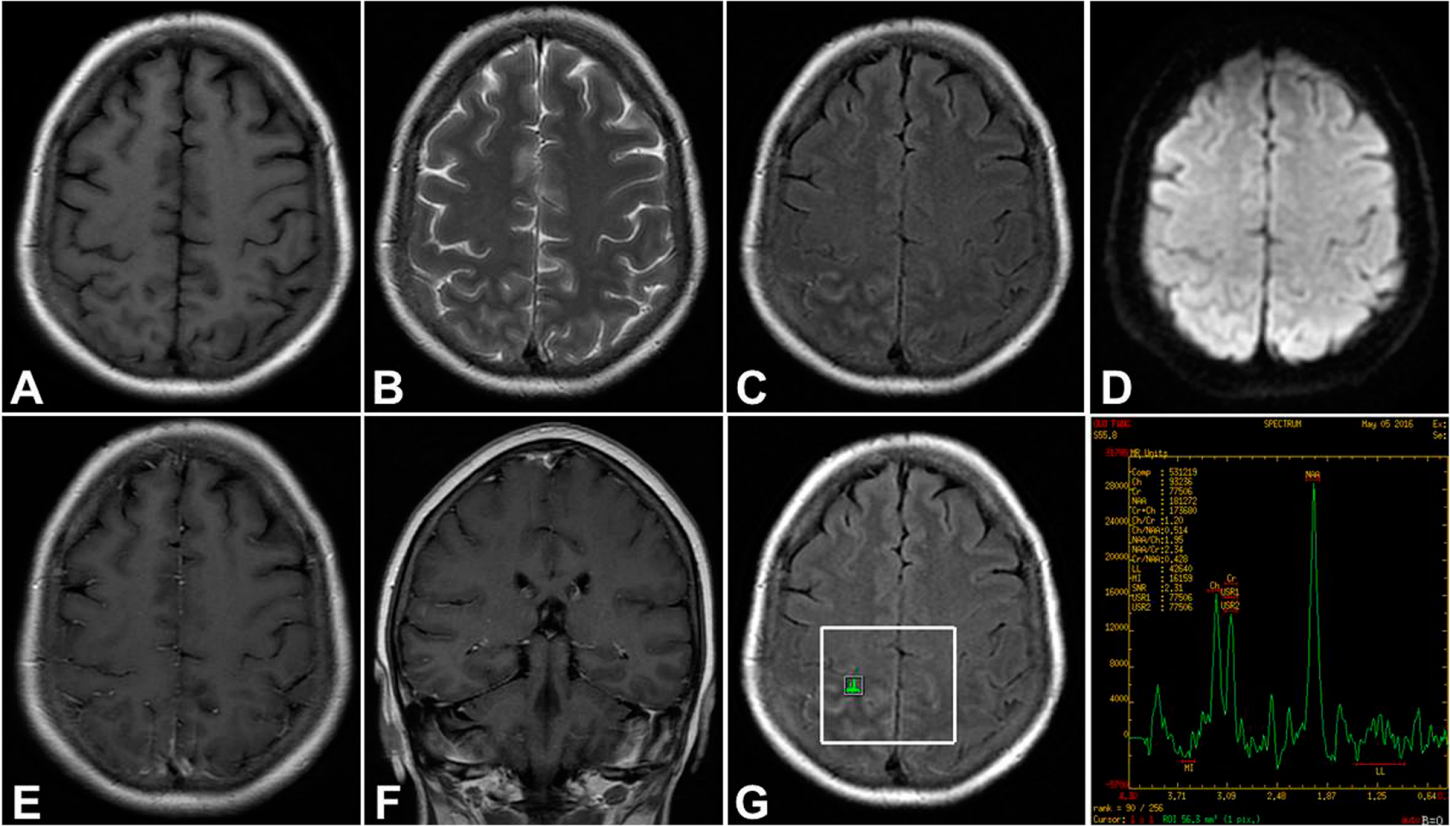
After treatment for 6 weeks, follow-up MRI showed nearly complete regression of lesion on *right* parietal lobe on T1-weighted (**a**), T2-weighted images (**b**), FLAIR (**c**), and still normal signal on DWI (**d**). MRI enhancement showed that the previous slim contrast enhancement almostly disappeared (**e**) and (**f**). MRS also showed normal NAA, Cr and Cho peak of previous lesion (**g**)

Based on a series of examination, the patient was diagnosed as having early undifferentiated connective tissue disease (UCTD). From the 5th hospital day, 10 mg of intravenous dexamethasone was administered for 14 days, and changed to oral prednisolone (30 mg/day) with gradual tapering (5 mg/month). At the same time, the patient was placed on maintenance therapy with azathioprine (50 mg/day). After treatment for 2 weeks, the symptom of left lower limb weakness was almost recovered. The patient was discharged and came in for regular visits. Follow-up MRI, performed at 6 weeks after the initial glucocorticoid therapy, showed much regression of lesion and enhancement on right parietal lobe. MRS showed normal NAA, Cr and Cho peak of previous lesion on the right parietal lobe compared to same region on normal left side (Fig. 3). Blood analysis showed normal erythrocyte sedimentation rate of 7 mm/h and C-reactive protein of 1.57 mg/L. The titer of antinuclear antibody decreased to 1:1000. Lumbar puncture and tests of the CSF showed normal intracranial pressure, glucose, chloride and protein level; the white cell count (WBC) was 2 × 106/L with normal percentage of lymphocyte 64.1%. The patient was continuously administrated with a low dosage of azathioprine and gradually tapering prednisolone, and followed up regularly.

## Discussion and conclusions

The concept of undifferentiated connective tissue disease (UCTD) was put forward for the first time by LeRoy in 1980, and considered to be the early stage of connective tissue disease (CTD), which could be developed into a specific kind of CTD, or maintained in undifferentiated state without progression [5]. After a long-lasting scientific debate, it is widely accepted that UCTD represent a distinct clinical entity with peculiar clinical findings; the proposed preliminary classification criteria for UCTD include: (1) at least one clinical manifestation of CTDs, (2) positive ANA results, and (3) disease duration of at least three years. However, the criteria may have limitation in making diagnosis, directing treatment and estimating prognosis of those patients with short disease duration no more than 3 years. In fact, although epidemiological data are not available in the literature, up to 50% of the patients with an undifferentiated CTD of less than one year of duration have been reported [6]. Recently, researchers have distinguished two different conditions of UCTD with variable disease course and prognosis, “stable UCTD” and “early UCTD”. Among the division, the so called “stable UCTD” refers to the condition that is up to the traditional criteria as described before and clinically stable over time only with necessarily mild therapeutic intervention. Moreover, the “early UCTD” refers to those patients with recent onset of symptoms and unclassifiable clinical picture which are very likely to progress into a definite CTD in the short time, and it has a crucial clinical importance in term of treatment decision making and disease monitoring [2].

Researches about stable UCTD have revealed that Raynaud’s phenomenon, artralgias/arthritis, skin rash and mild cytopenias are the most frequent onset manifestations; the serological autoantibody profile frequently presents single specificity with ANA positivity highly ranging from 58–90% [1, 7–9]; and after low-dose corticosteroids and immunosuppressive drugs treatment, 70% stable UCTD patients will be maintained in undifferentiated state without progression [6]. Among these stable UCTD patients, severe organ involvements such as heart, renal or neurological manifestations were rarely reported. As far as we know, only 3 cases of CNS involvement in stable UCTD have been reported. Among these cases, two of them were hypertrophic cranial pachymeningitis (HCP), and the other one was reversible posterior leucoencephalopathy syndrome (RPLS). After symptomatic treatment, low-dose corticosteroid or immunosuppression treatment such as azathioprine or methotrexate, all the patients’ symptoms and radiologic abnormalities of brain were improved [10–12]. However, up to now, there is still lack of definite researches about clinical course, prognosis and organ involvement of early UCTD.

In ours case, the increased erythrocyte sedimentation rate, C-reactive protein and titer of antinuclear antibody suggested an active systemic autoimmune disease, but the patient did not meet the existing classification criteria for a certain connective tissue disease, and the disease duration was no more than 3 years. Therefore, the patient was diagnosed as having early UCTD. The lesion on right parietal lobe, presenting hypointensity on MRI T1, hyperintensity on T2 and high signal on FLAIR, was responsible for the onset symptom of left lower limb weakness. The patient declined a cerebral biopsy, but according to the normal intracranial angiogram, the results of CSF study, MRI enhancement and MRS examination, we could exclude the possibility of stroke, cerebral venous sinus thrombosis, NMOSD, MS, autoimmune encephalitis, intracranial infections, and malignant tumors as cause of the lesion. Moreover, examinations in the CNS showed slightly elevated white cell count with increasing percentage of lymphocyte in CSF, and slim contrast enhancement along the surface of swollen gyrus lesion in MRI. Thus, we presumed CNS involvement in this early UCTD patient. After treatment of low-dose corticosteroid and azathioprine, the symptom of left lower limb weakness was almost recovered, abnormalities of immunologic tests and neuroimaging were also greatly improved within a short duration. To our knowledge, the case we described here is the first report of CNS involvement as onset manifestations in a patient with early UCTD.

In summary, CNS involvement may be the onset symptom in early UCTD, and must be recognized early in the presence of systemic blood exam alterations such as high ESR, CRP and ANA, because of good prognosis with prompt and adequate treatment. Diagnosis is difficult as all causative factors must be excluded, and cerebral biopsy is not always feasible. Low-dose steroid and immunosuppressive therapy seems to be effective to the CNS damages in early UCTD, and long-term follow-up is also necessary to investigate the prognosis of early UCTD and CNS involvement.

## Additional file

Additional file 1: Oligoclonal bands are negative and shown below (CSF: cerebrospinal fluid, S: serum, C: positive control). (JPG 1452 kb)

## Abbreviations

ANA: Antinuclear antibody
CNS: Central nerve system
CSF: Cerebrospinal Fluid
CTD: Connective tissue disease
MRI: Magnetic resonance imaging
MS: Multiple sclerosis
NMOSD: Neuromyelitis optica spectrum disorder
UCTD: Undifferentiated connective tissue disease

## References

[1] Mosca M, Neri R, Bombardieri S (1999). Undifferentiated connective tissue diseases (UCTD): a review of the literature and a proposal for preliminary classification criteria. Clin Exp Rheumatol.

[2] Mosca M, Tani C, Vagnani S (2014). The diagnosis and classification of undifferentiated connective tissue diseases. J Autoimmun.

[3] Bodolay E, Csiki Z, Szekanecz Z (2003). Five-year follow-up of 665 Hungarian patients with undifferentiated connective tissue disease (UCTD). Clin Exp Rheumatol.

[4] Mosca M, Tani C, Carli L (2013). Analysis of the evolution of UCTD to defined CTD after a long term follow-up. Clin Exp Rheumatol.

[5] LeRoy EC, Maricq HR, Kahaleh MB (1980). Undifferentiated connective tissue syndromes. Arthritis Rheum.

[6] Mosca M, Tani C, Bombardieri S (2008). A case of undifferentiated connective tissue disease: is it a distinct clinical entity?. Nat Clin Pract Rheumatol.

[7] Mosca M, Tani C, Talarico R (2011). Undifferentiated connective tissue diseases (UCTD): simplified systemic autoimmune diseases. Autoimmun Rev.

[8] Mosca M, Tavoni A, Neri R (1998). Undifferentiated connective tissue diseases: the clinical and serological profiles in 91 patients followed for at least 1 year. Lupus.

[9] Mosca M, Neri R, Bencivelli W (2002). Undifferentiated connective tissue disease: analysis of 83 patients with a minimum follow up of 5 years. J Rheumatol.

[10] Lampropoulos CE, Zain M, Jan W (2006). Hypertrophic pachymeningitis and undifferentiatedd connective tissue disease: a case report and review of the literature. Clin Rheumatol.

[11] Kim JH, Joo YB, Kim J (2010). A case of hypertrophic cranial pachymeningitis presenting with scleritis in a patient withundifferentiated connective tissue disease. J Korean Med Sci.

[12] Singh S, Balakrishnan C, Mangat G (2006). Reversible posterior leucoencephalopathy syndrome in a patient with undifferentiated connective tissue disease. Scand J Rheumatol.

