# Supplementary figures and images for: Intracranial lesion as onset symptom in a patient with early undifferentiated connective tissue disease: a case report
Source: BMC Neurol. 2017 May 5;17:85. doi: 10.1186/s12883-017-0868-4 (PMC5420101; doi:10.1186/s12883-017-0868-4)

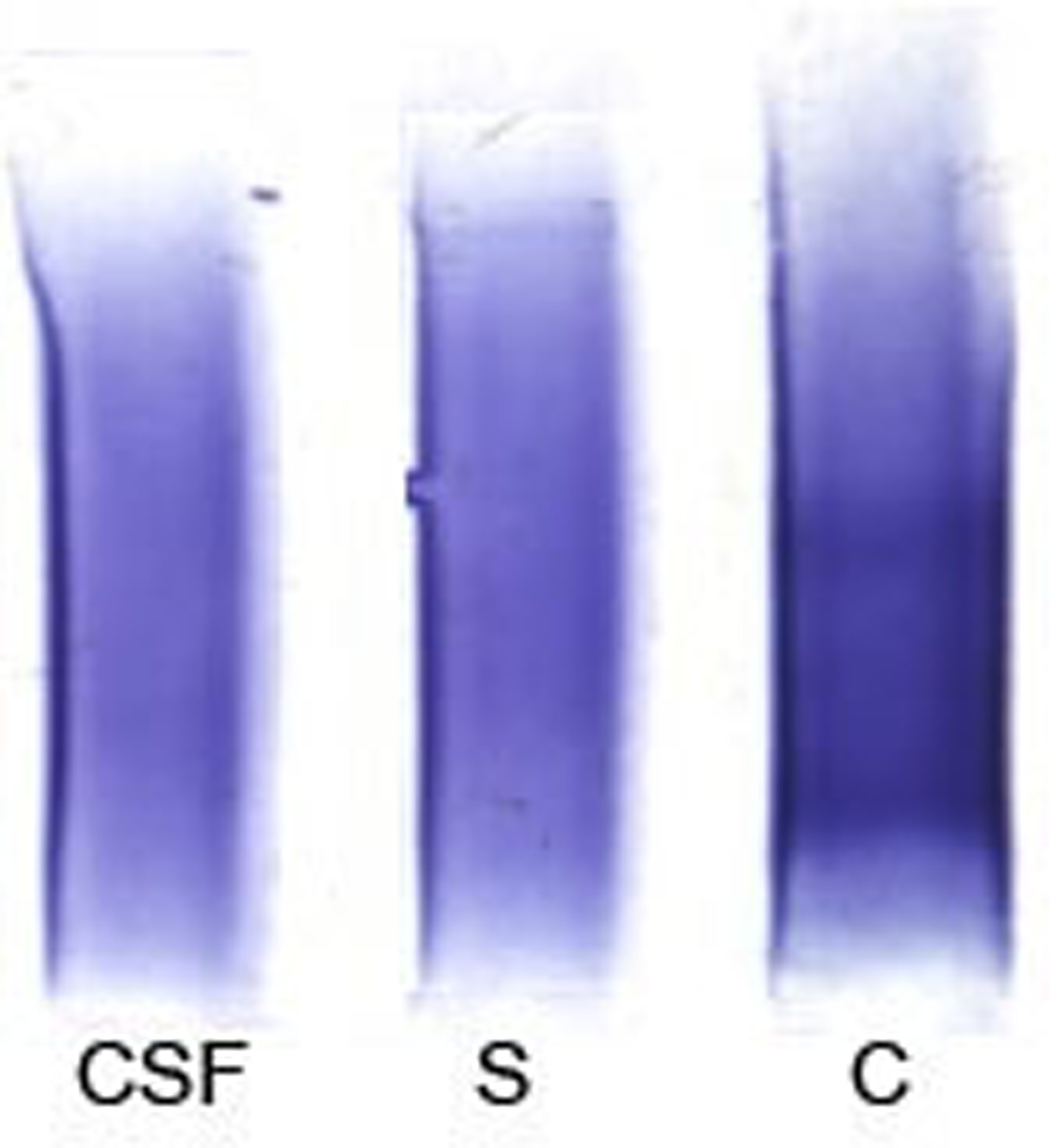

Supplement: Additional file 1: — Oligoclonal bands are negative and shown below (CSF: cerebrospinal fluid, S: serum, C: positive control). (JPG 1452 kb) [file 12883_2017_868_MOESM1_ESM.jpg]
